# Active Aging Promotion: Results from the *Vital Aging* Program

**DOI:** 10.1155/2013/817813

**Published:** 2013-02-07

**Authors:** Mariagiovanna Caprara, María Ángeles Molina, Rocío Schettini, Marta Santacreu, Teresa Orosa, Víctor Manuel Mendoza-Núñez, Macarena Rojas, Rocío Fernández-Ballesteros

**Affiliations:** ^1^Department of Psychology, Madrid Open University (UDIMA), Collado Villaba, 28400 Madrid, Spain; ^2^Institute for Advanced Social Studies, Spanish National Research Council, 14004 Córdoba, Spain; ^3^University Program for Older Adults (PUMA), Autonomous University of Madrid, Cantoblanco Campus, 28049 Madrid, Spain; ^4^Department of Psychobiology and Health, Autonomous University of Madrid, Cantoblanco Campus, 28049 Madrid, Spain; ^5^University of La Habana, 11600 La Habana, Cuba; ^6^Gerontology Research Group, National Autonomous University of Mexico, FES Zaragoza Campus, 09230 Mexico City, DF, Mexico; ^7^Older Adults Program, Catholic University of Chile, Santiago Metropolitan Region, Santiago, Chile

## Abstract

Active aging is one of the terms in the semantic network of aging well, together with others such as successful, productive, competent aging. All allude to the new paradigm in gerontology, whereby aging is considered from a positive perspective. Most authors in the field agree active aging is a multidimensional concept, embracing health, physical and cognitive fitness, positive affect and control, social relationships and engagement. This paper describes *Vital Aging*, an individual active aging promotion program implemented through three modalities: Life, Multimedia, and e-Learning. The program was developed on the basis of extensive evidence about individual determinants of active aging. The different versions of *Vital Aging* are described, and four evaluation studies (both formative and summative) are reported. Formative evaluation reflected participants' satisfaction and expected changes; summative evaluations yielded some quite encouraging results using quasi-experimental designs: those who took part in the programs increased their physical exercise, significantly improved their diet, reported better memory, had better emotional balance, and enjoyed more cultural, intellectual, affective, and social activities than they did before the course, thus increasing their social relationships. These results are discussed in the context of the common literature within the field and, also, taking into account the limitations of the evaluations accomplished.

## 1. Introduction

The concept of aging well as a scientific field dates back to the early 1960s, within the context of the World Health Organization (WHO), when Roth highlighted the importance of health promotion and illness prevention throughout the life span, and especially in old age [1]. Most importantly, in the 1980s, one of the pioneers in the field of aging well, Fries, would stress the modifiability and plasticity of the human being throughout life and into old age, listing non-modifiable negative conditions associated with age and their correspondence with modifiable preventive factors [2–4]. Recently, Christensen, Doblhammer, Rau, and Vaupel noted how, since the 1950s, mortality after age 80 years has steadily fallen, with life expectancy lengthening almost in parallel with best practices over the last 150 years [5] and they showed evidence that human senescence has been delayed by a decade [6] strongly associated with “healthy best practices.” 

In fact, the aging revolution is the result of falling mortality rates and the corresponding increase in life expectancy. But, these changes in the population are due not only to biomedical advances, but also to the exponential development of human society across history: compulsory education, economic growth, the extension and democratization of the improvement of life conditions, better healthy practices, extended scientific knowledge, and so forth, have all made their contributions to this revolution. At the same time, we have seen the emergence of the active aging paradigm [7, 8].

In the WHO document *active ageing*. *A policy framework*, the determinants of active aging posited were mainly population-based: Economic, Social, Environmental, and Health and Social Services, suggesting that the responsibility for active aging lies with the public sector, through public health programs and social policies [9]. The implementation and evaluation of such programs are necessarily long-term, and therefore highly complex. As Christensen et al. show, one way of evaluating “best practice” in health is through the association of such practices with population-based indicators such as mortality, or life expectancy, or even disability-free life expectancy, healthy life expectancy, or quality-adjusted life years [5]. 

Even so, as stressed elsewhere, not only it is important to promote active aging from a population-based point of view, it is also relevant to do so from an individual perspective. Aging well is not a random phenomenon: the individual is an agent of his/her own aging process, and the capacity for aging actively comes not only from sociopolitical actions, but also through decisions taken by individuals themselves. Thus, among the determinants posited by the WHO, two types of individual-based factors can be found: Behavioral (lifestyles) and Personal (both biogenetic and psychological) [10].

Active aging is a multidisciplinary concept (also called successful, productive, or optimal aging), and cannot simply be reduced to “healthy aging,” needing, rather, to take into account protective behavioral determinants (protective life styles and the prevention of risk factors) [10–12]. Moreover, a definition of aging well must include other psychosocial factors, such as cognitive and mental functioning, positive mood, sense of control, active coping styles, and social participation and engagement. Promotion and education in relation to these factors through psychosocial initiatives extending the encouragement of healthy lifestyles (such as physical activity or good nutrition) to other aspects, such as memory training, stress management, self-efficacy coaching, or training in prosocial behavior, would appear to represent a step forward in the promotion of active aging. Supporting literature of those aspects will see shortly listed when our four domains model will be presented.

This paper deals with a set of psychosocial and educational interventions called “*Active Aging*” with various formats (*Life Course, Multimedia*, and e-Learning) for the promotion of active aging at the *individual level*—that is, *without* modifying any of the posited determinants at the population-based level (income, macrosocial and environmental conditions, or health and social services).

Here we consider three programs, all of which have been implemented at the Autonomous University of Madrid (UAM; Spain), and the last one also in other three Latin American Universities: *Vital Aging* life, *Vital Aging* multimedia and *Vital Aging* e-Learning. 

## 2. *Vital Aging* Program

Here we provide a brief presentation of the *Vital Aging* Program summarized from other published materials [10, 12–17].

### 2.1. Basic Principles

Underlying the *Vital Aging *is a set of theoretical assumptions.

1. There are major differences in forms of aging (normal, optimal, and pathological), and there is empirically-based knowledge about how to age well [4, 18].
2. This diversity across the life course is not random. External circumstances are crucial to the aging process, but the individual is also an agent of his or her own aging process [19].
3. Plasticity is a property of the Central Nervous System, but also of the human organism. Plasticity, though subject to certain limitations, remains throughout the life span and into old age. Over the course of life, plasticity is expressed through learning and modifiability [7, 20, 21].
4. Selection, Optimization, and Compensation are adaptive mechanisms found within the aging process; knowledge-based pragmatics, high motivation, and technology can compensate decline [7].

### 2.2. A Four-Domain Model for the *Vital Aging* Program

Underpinning the content of the *Vital Aging* program is a 4-domain model of aging well posited by Fernández-Ballesteros, [10, 22] whereby active aging is defined as the lifelong adaptation process of maximizing health and independence; physical and cognitive functioning; positive affect and control; and social engagement [10].

**Figure 1:**
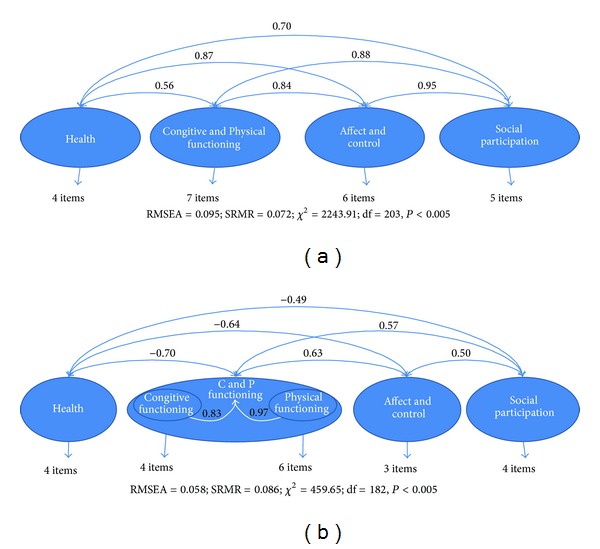
Structural Equations Modeling of four-domains model of ageing well: (a) from lay conceptalizations (*N* = 1,189), and (b) from ELEA PROJECT multimethod data base (*N* = 458).

As shown in Figure 1, this four-domain model of aging well has recently been tested by Fernández-Ballesteros et al. [23] through Structural Equation Modeling, with data both from our cross-cultural project on lay definitions of aging well provided by older adults from 7 Latin American and 3 European, [24–26] and from the ELEA research project (Longitudinal Study of Active Aging) [27].

As far as the four domains of active aging are concerned, they are not only based on Structural Equation Modeling using empirical data (from lay definitions and research findings), but also strongly supported by the scientific literature. Although, this is not the place to present all such evidence (for a review, see: Fernández-Ballesteros, 2008), [10] let us consider some examples.

**(i) Behavioral Lifestyles**

(1) Regular physical exercise reduces mortality risk by about 35% (e.g., Healthy Aging Longitudinal European study) [28, 29]. (2) Elders with healthy behavioral life styles show *four times less *disability than those who smoke, drink too much, do not exercise, and are obese. Moreover, in those with good behavioral habits the onset of *initial disability was postponed by 7.75 years* [30]. (3) Netz et al. carried out the most recent meta-analysis of those studies linking physical activity to mental health and well-being. Studies with older adults shows that effect sizes for physical exercising treatment groups were almost 3 times as large as the mean for control groups [31]. (4) Mediterranean diet (low intake of saturated and trans fat and high consumption of fruit and vegetable) is stronger related to survival and life expectancy [32–34]. (5) This type of diet decreases coronary mortality about 40% and all causes of mortality about 20% [35, 36].

**(ii) Cognitive Activity and Training**

(1) More frequent cognitive activity in everyday life is associated with a reduction of approximately 19% in annual rate of cognitive decline, and is also a protective factor against dementia [37, 38]. (2) The effects on cognitive functioning of cognitive training are of a magnitude equivalent to the decline expected in elders without dementia over a period of 7 to 14 years, though longer follow-up study is required [39]. (3) Memory training yields effects sizes of 0.75 SD, by comparison with 0.40 as a practice effect, in both objective memory tests and subjective memory functioning [40, 41]. (4) A meta-analysis carried out by Colcombe and Kramer with 18 intervention studies examining the effects of physical fitness training on cognitive functions yielded robust effects for several measures of cognitive functioning [42]. In sum, all these progresses support not only a more complex view of cognitive functioning across life span but a new panorama in which effective cognitive trainings and intervention can optimize cognitive functioning, compensate intellectual losses and declines or even palliative cognitive impairment (for a review see: Hertzog et al, 2009) [41].

**(iii) Positive Affect, Coping, and Control**

(1) *Positive Affect *reduces mortality in older individuals. The benefits of positive affect can be observed in conditions as diverse as stroke, re-hospitalization for coronary problems, the common cold, and accidents; highly activated positive emotions were associated with better functioning of cardiovascular, endocrine, and immune systems [43]. A positive attitude towards life may help us avoid becoming frail. For those reporting positive affect 7 years earlier, the chance of becoming frail fell by 3%, while the chances of having better health outcomes, greater functional independence increased, as did survival rates. The authors conclude from these finding that positive affect is protective against functional and physical decline in old age as well as negative affect such anxiety are requiring coping and management [44]. The most important conclusion emerging from coping and aging literature is that although there is a broad evidence about the stability of coping behaviour across life span, authors distinguish specific positive coping skills in old age which can be trained and promoted [45, 46]. (3) Sense of Control and Self-efficacy. Older adults with a high sense of control are better off on many indicators of health and well-being and those who have a lower sense of control may be at increased risk for a wide range of negative behavioral, affective, and functional outcomes, including higher levels of depression, anxiety, and stress, use of fewer health protective behaviors (e.g., exercise) and compensatory memory strategies (e.g., internal or external memory aids), and have poorer health and memory functioning. Also, the sense of control is a powerful psychosocial factor that influences well-being and it is a good predictor of healthy and active aging; finally, sense of control can be trained as has been largely tested [47–49]. Among control concepts, self-efficacy is perhaps the best well-known construct in successful ageing literature. In the last twenty five years self-efficacy has been searched through cross-sectional, longitudinal and experimental designs [19]. Self-efficacy beliefs are strongly related with successful aging, firstly because they contribute to perceive age related situations not as threats but as challenges; secondly, because they support to individual to remain committed in selected goals and, finally, because self-efficacy perceptions have a synergic power with other factors for enhancing outcomes [10]. (4) Self-stereotypes or self-images about aging reduce the risk ratio of .87 (*P* < .001). Persons with positive images about aging (assessed 25 years earlier) lived 7.5 years more than those reporting poor self-perception of aging at baseline. One aspect of the positive self-perception of aging measure, risk of dying, fell by 13% [50, 51].

**(iv) Social Functioning and Participation**

(1) The association between social relationships and the prevalence and incidence of and recovery from disability has been well established [52]. (2) Research results have shown a strong and robust cross-sectional association between social engagement and disability, more socially active persons reporting lower levels of disability than their less active counterparts [53]. (3) There is empirical evidence that social activity and participation improve cognitive functioning [54]. (4) Results have shown that the protective effects of social engagement diminish slowly over time [55].

In summary, there is strong support for these four domains of active aging on which the *Vital Aging* Program is based (for a review, see Fernández-Ballesteros, 2008) [10].

### 2.3. *Vital Aging* Program Versions

The starting point was the *Vital Aging* course (Vivir con Vitalidad) developed in 1996 at the autonomous university of Madrid (UAM), developed by Fernández-Ballesteros as an open life course. Since 1996, several editions of the *Vital Aging* course have been run; a multimedia version and an e-Learning course have also been developed. Let us now describe these three programs.

**(i) *Vital Aging* L (1996–2003)**

Organized as a continuing education course at the UAM, it consists of 20 thematic units over 70 hours (3 hours per session, 2 sessions per week). Trainers are experts in a variety of subjects, teaching in highly practical way and supported by a basic text (drawn up by Fernández-Ballesteros) [12]. All sessions have a similar structure: (1) the trainer makes a general presentation of the content in question, talking about the supporting evidence on each unit; (2) a pretest for the particular behavioral or psychological characteristic (diet, physical exercise, self-efficacy, pleasant activities, etc.) is administered; (3) practical strategies for better aging are described and reviewed, and exercises are performed; (4) at the end of the class a post-test is administered, and the results are discussed; (5) finally, the trainer makes some concluding remarks (for a summary, see Table 1).

**(ii) Vital Ageing M**

The *Vital Aging*-L was transformed in to the *Vital Aging* multimedia course developed under the auspices of the European Commission, as a Socrates-Minerva Program, by a Consortium made up of UAM (Spain), Nettuno (Italy) and the University of Heidelberg-Institute of Gerontology (Germany), and with the cooperation of the Open University (UK). *Vital Aging*-M consists of 48 hours of video lessons grouped in 20 Thematic Units with supporting materials on the Internet. Each Unit comprises 2 to 4 hours' video-lessons taught by European experts from Germany, Italy, and Spain (so far the program is available only in Spanish). Although, at the very beginning *Vital-Aging*-M program was broadcasted through the Italian TV-Chanel 2, all our evaluation studies were based on the administration of those video-lessons in the class-room by a trained tutor who is in charge of all equipments, the distribution among participants of the supporting materials, and the assessment instruments for each video-lesson. Participants follow all sessions of each lesson, fill out the instruments, and work with the material distributed present in the video-lesson. Each lesson lasts approximately 2 hours, with a break of 15 minutes between sessions. Lessons have the same structure and content as in *Vital Aging*-L and e-Learning versions (Table 1).

**(iii) *Vital Aging* e-Learning**

The program was supported by the UAM-Santander Inter-University Cooperation Program for Latin America (2010–2012), with the main goal of developing an e-learning methodology for senior citizens' university programs (PUM-e). In the first step, a pilot format of the program was implemented at UAM and the Catholic University of Chile, and subsequently assessed. Based on this pilot study, *Vital Aging* materials designed to be used on via Internet by Fernández-Ballesteros (http://www.vivirconvitalidad.com/) were adapted cross-culturally with the contribution from the three Latin American universities. Several changes were made to obtain an e-learning format that could be implemented through the Learning management System, LMS-Moodle Platform. Finally, the program was launched at the four participating universities: UAM, Catholic University of Chile, La Habana University (Cuba) and the National Autonomous University of Mexico. The *Vital Aging* e-Learning program requires around 65 hours of work, and was run over a period of three months. Students had a set of learning resources as follows. (1) Self-evaluation: in order to give the student a base-measure of his/her performance in each basic unit, a questionnaire is filled out, the responses being checked automatically. This self-evaluation is useful for making students aware of their status in relation to each work module. (2) Readings: these provide useful, relevant, and proven information on the various topics addressed by the program. (3) Activities: two types of activity are involved, those used to verify self-knowledge related to the readings and those that serve for planning changes to be incorporated into daily life. (4) Forums: these are designed to promote discussion among students (including inter-country discussion) and the exchange of views about the various topics taught on the course. (5) Tutorial: the course offers the assistance of a Virtual tutor, who provides information about the execution of the task throughout the course and resolves any doubts that may arise regarding the materials and program content, and an On-site tutor, who deals with the technical difficulties that may arise on using the Moodle platform.

In order to allow comparisons of our materials and methods, Tables 1 and 2 show the procedures followed and the materials (domains, units, contents as well as the assessment and practice) for the three *Vital Aging* versions. 

### 2.4. *Vital Aging* Program Hypothesis and Objectives

Our general hypothesis was that after *Vital Aging* programs, experimental individuals, in comparison to pre-test and controls, significantly, will attain the objectives of the program as measured by the instrument administered.

Objectives are the following: (a) to teach basic knowledge how to age well; (b) to promote healthy behavioral lifestyles; (c) to train strategies for optimizing cognitive functioning and compensating potential decline; (d) to optimize positive affect and emotion, promoting control and coping styles; (e) to promote social relationships and participation throughout the life course using new technologies.

### 2.5. Teaching Materials

“*Vital Aging*-L,” “*Vital Ageing*-M,” and “*Vital Aging*-e” are multidimensional courses based on the same four-domain model of active aging. Therefore, materials (units, lesson content, assessment tests, and tasks for practical work) were developed on the basis of these four domains. The *Vital Aging* e-Learning version is less extensive than *Vital Aging* Life and Multimedia, but after a general introduction, the four domains are addressed.  Table 2 shows a summary about Domains and Units, together with examples of Context and Assessment tests and Practice tasks for the three versions of *Vital Aging*.

## 3. Evaluation Studies on *Vital Aging* Programs

Four evaluation studies have been carried out on Active aging programs: following Scriven, formative evaluations were conducted at the beginning of both the *Multimedia* and *e-Learning* versions; [56] also, several summative or outcome evaluations were carried out for the *Life* and *Multimedia* programs; finally, since the *e-Learning* version is quite new, a pilot outcome evaluation is reported. The formative evaluation focused on the materials used, on participants' views about the course and about changes that occurred, and finally on their satisfaction. Summative or outcome evaluations were performed on the basis of quasi-experimental/quasi-control designs (pre-post with control group), in order to test the objectives of the *Vital Aging* programs; that is, the extent to which they gave rise to expected changes [57].

### 3.1. Evaluation Studies

A first evaluation of *Vital Aging* M was carried out during 2002 and 2003. This study involved a comparison between *Vital Aging*-M participants living in residential facilities (*N* = 13, mean age = 79.3) and others living in the community (attending senior citizens' clubs; *N* = 44, mean age = 69.9). The control group was recruited in the same contexts, from those doing other activities (*N* = 31, mean age = 74.2). After 6 months, a follow-up of those participants living in the community was carried out. Participant characteristics, procedures, materials, and results are reported elsewhere [15].

In the second study, the *Vital-Aging*-M program (*N* = 25; Mean age = 69.5) was compared with *Vital aging* L (*N* = 28, mean age = 67.84). The two programs were also compared under similar quasi-experimental conditions to a control group (*N* = 37, mean age = 65.6). Control participants were recruited from among those attending other regular activities at the Community Centre. Participant characteristics, procedures, materials, and results are reported elsewhere [13, 14].

In our third study, participants were 115 people aged over 54. Of these, 73 had attended five different editions of the *Vital Aging-M* program (mean age = 62.56, 52.2% women) and 42 had not attended the program (though they were on the waiting list), though they filled out the same questionnaire at the same point; these latter participants made up the control group (*N* = 42, mean age = 62.29; 57.5% women) [17].

Finally, our fourth evaluation study refers to the *Vital Aging-e learning *program recently implemented (January–April 2012) and evaluated. Participants filled out the Formative and Summative protocol; only Formative results are going to be reported here, since summative evaluation is not yet finished; only some provisional data from the Spanish subsample will be reported. Sample characteristics of the four studies are summarized in Table 3.

In order to operationalize objectives two Protocols were set up with different assessment instruments administered during the program. Formative Evaluation Protocol covers the following variables: achievement tests (with the aim of checking whether there were effects on knowledge about the course units); appraisal of lessons (referring to aspects of the lesson itself); self-perceived changes (about expected changes in behavior and psychological characteristics), and satisfaction with the course. Based on the program objectives, Summative Evaluation Protocol contains a series of questions related to the following dependent variables: Views of aging (for testing changes in stereotypes and self-perceptions on aging), Activities performed (leisure, social, intellectual, cultural, etc.), Physical exercise and Nutrition (in order to assess lifestyles), Health problems, Social relationships (frequency, quality and satisfaction), and Life satisfaction. In our third study the following variables were also included: subjective memory, mnemonic strategies, memory appraisal, self-efficacy for aging, and positive and negative affect.

For each study, statistical analyses were carried out separately for each group, since the interest reside in observing to what extent they showed similar patterns of results, means obtained before and after each version using a repeated-measures *T* test were performed. We also compared the pre- and post-test means of the experimental groups with that of the control group. Covariant analyses were performed in order to test potential effects of age and gender on results.

## 4. Summary of Results 

### 4.1. Formative Evaluation

#### 4.1.1. Achievement Test

First of all, based on the lesson's readings, trainers drew up ten questions for each lesson. Internal consistency and difficulty levels were assessed. In general, *Vital Aging-M* participants scored at least 50% correct answers in all achievement tests. Lessons yielding the highest scores were those on “Positive thinking,” “Coping with stress,” and “Sexual relationships: Beyond genitality.” Those yielding the lowest scores (never lower than 50%) were “Creative aging,” “Some basic facts about memory skills,” and “Nutrition and health”. These results were very helpful for improving lesson materials, since they allowed us to clear up some confusing aspects. 

#### 4.1.2. Appraisal of Lessons

The most positively rated lessons of *Vital Aging-M *were “Aging well” and “Taking care of your body” (both with all elements rated as equal to or above the mean score), while the lowest-rated were some of the lessons originally taught in a language other than Spanish and later translated and dubbed. All of these were rated below the mean score. Since there is a strong relationship between level of knowledge and rating of the different details of the lessons, several analyses were performed to identify which elements of the lessons are most closely related to the general level of achievement. The variable that best predicts the level of knowledge attained in a lesson is “Teacher's clarity of presentation” (*r* = .607), followed by “Interest of the exercises” (*r* = .601), “Usefulness of the exercises” (*r* = .545), and “Satisfaction with the lesson” (*r* = .527). In any case, it should be stressed that knowledge achievement correlates positively and significantly (*α* = 0.05) with the opinions expressed. 

Also, the appraisals of the lessons results were very helpful for improving materials. 

#### 4.1.3. Self-Perceived Changes

At the end of the Course, participants reported the degree of change they perceived, with regard to each of the units involved. The results showed that “Enjoying life in general,” “Thinking positively,” “Improve memory,” “Feeling self-efficacy,” and “Pleasant events and well-being” were the domains in which participants perceived the most positive changes. On the other hand, “A new system of communication: Internet,” “Sexual relationships,” “Creative aging,” and “Improving family and social relationships” were the areas in which they reported minor changes.

As regards *Vital Aging e-Learning* participants, 62% reported that they had made quite a few of or many of the changes suggested in the course. Seventy-six per cent (76%) of these changes referred to emotions (positive thinking, managing stress, enjoying life in general, feel effective, enjoyable activities); 73% were related to cognitive functioning (training the mind, memory, wisdom); 69% concerned social relationships (relations with family and friends); 51% referred to lifestyles (body care, nutrition, exercise); and finally, 48% concerned participation (volunteering and Internet use). Regarding the intention to introduce changes in the future, 59% reported that they are going to incorporate some changes proposed in the program, and 35% that they would plan to incorporate very many changes. 

#### 4.1.4. Satisfaction with the Course

More than two-thirds of the *Vital Aging-M* course participants found the course very interesting, and no one reported low or none interest. The course met “fairly well” or “totally” the expectations of 98.8% of the participants, and 96.7% considered that the knowledge learnt had been useful or would be useful in the future. The difficulty of the course was considered low by 45.1%, while for 82.9% its content was already partially known, and 79.3% felt they had learned a great deal. General level of satisfaction was high (78.8%), and there were no participants with low satisfaction. The most negative aspect in relation to this evaluation concerns the fact that the participants scarcely consulted the reading materials available on the homepage, consulted the tutor by interview very little or not at all (78.5%), and made practically no use of the Internet at all (89.9%).

As far as *Vital Aging e-Learning* participants are concerned, 95.8% reported that the course was quite or very interesting; 80.6% considered that they performed all the program tasks proposed; 94% considered that their expectations about the course were sufficiently met; and 96% reported that the contents of the course were very helpful for improving daily living. Regarding the level of difficulty of the course, 59.7% considered the course was not easy or not very difficult, 33.3% reported that the difficulty of the program was low, and only 6.9% perceived a high level of difficulty. Regarding satisfaction about the course, 77.5% reported that they were highly satisfied, and only 5.6% said that their level of satisfaction with the course was low. Finally, we asked participants to rate, on a scale of 1 (none) to 10 (maximum), to what extent the program would help them to grow-upas persons, the average score being 8.36 (SD = 1.93).

In summary, our formative evaluations served to improve our materials, but they also provided a subjectively positive view of the programs. Even so, our objective was not only to promote well-being, but also to produce changes in several target behaviors related to active aging, thus let introduce those outcomes.

### 4.2. Summative Evaluation

First of all, it should be emphasized that our experimental and control groups did not significantly differ in the pretest with regard to the dependent variables and both sociodemographic variables, age, and gender do not have influences in any of the dependent variables. In comparisons between pre- and post-test measures in the experimental groups and between experimental and control post-test measures, significant differences were yielded in the following variables in the expected direction.

1. Views of aging: Those participating in the *Vital Aging* programs were assessed (both *Vital Aging*-M and *Vital Aging*-L, and those living in residences and in the community) had a significantly better view of aging after the course, and also they considered themselves more efficient for facing the aging process. No significant pre-test/post-test differences were found in our third study for views of aging.
2. Activity level: After the implementation of both *Vital Aging* programs assessed (Life and Multimedia), participants from both contexts (Community and Residence) reported higher frequency of cultural, intellectual and social activities while not changes were found among controls.
3. All those participants living in the community attending *Vital Aging-M* or *Vital Aging-L* did significantly more *physical exercise* and significantly improved their *diet* after the course. These positive effects were not found in those participants living in residential settings.
4. Regarding *Vital Aging-M*, no significant pre-test/post-test differences were found in our experimental groups in either context (residence or community) for the social relationships measures. Only participants in *Vital Aging-L* and those attending the program in the third study yielded positive results, reporting significant increases in the frequency of their social relationships.
5. With respect to life satisfaction, participants in *Vital Aging-M* living in the community reported greater differences after the program in the first and the second studies. Nevertheless, no differences were found in those participants living in residences or in those participating in the same *Vital Aging* program in our third study.
6. In the follow-up carried out for our first study (after 6 months), all pre-post differences in the experimental group were maintained, but, as predicted, positive changes were found in health for the community group.
7. All of these differences remained significant when the effect of age was controlled.
8. In the third study, after attending *Vital Aging-M* participants reported better memory and more use of mnemonics, improved their hedonic balance, experienced fewer negative emotions, and increased the frequency of their social relationships.
9. Regarding *Vital Aging e-Learning*, preliminary results obtained in the Spain subsample indicate that following the program, participants reported greater emotional balance, and higher leisure and productive activities. All of these results are consistent with the other *Vital Aging* versions.

## 5. Discussion

Although some findings are not totally consistent (mainly for life satisfaction and social relationships), *Vital Aging* programs yield quite encouraging results. Participants enrolled on *Vital Aging-Life *and *Multimedia* had a better view of aging, in accordance with what was presented in the program units. Likewise, they more frequently enjoy cultural, intellectual and social activities than they did before the course. With the exception of participants living in residences, all the experimental groups increase their physical exercise and significantly improve their diet. The results are in accordance with those from the literature on programs promoting physical exercise and healthy diet, and are similar to previous results about activity level [28, 58, 59].

Nevertheless, these positive results on physical activity and diet were not found in the Residence group. Therefore, it can be concluded that *Vital Aging-M* had much more impact in the community than in institutions. However, although these differences in favor of our participants living in the community could be attributed to the fact that they have much less control over their institutional context than those living in the community, it should also be attributed to age, since those living in residential settings participating in our study are older than those living in the community (a general pattern for residential settings in Spain). This pattern is in accordance with findings from the general literature in the field of programs implemented in institutions and in studies comparing implementations in the community and in residences, as reported by Dwyer et al., among others [60]; nevertheless, any conclusions would be premature, since the numbers of participants in our residence group was very small.

Satisfaction or well-being is one of the targets for most programs promoting active aging. Nevertheless, while the measure in our first Summative Evaluation Protocol was life satisfaction, *Vital Aging-M* yielded a significant increase in life satisfaction only in the community group (not in residences) in the first and second studies, with no differences found in the third study. In sum, we failed to obtain changes in life satisfaction in two of our three studies. It should also be noted that when we introduced more specific variables of affect, in our third study, the *Vital Ageing-M *participants reported more positive emotional balance; that is positive affect is significantly higher than negative in the same direction as found in other studies [61, 62]. Although much more research is necessary, our results point to the stability of life satisfaction construct do not make it as a sensitive variable for evaluation purposes, as also reported by several other authors; [63] therefore, more specific measures of satisfaction and well-being must be used.

Participants in the *Vital Aging*-M program in the third study (the only study in which we used these variables) also reported better perceived health and significantly improved their appraisal of their memory, reporting the extensive use of mnemonics, improved their hedonic balance by experiencing fewer negative emotions, and increased the frequency of their social relationships. All of these results could be attributed to two circumstances. First, after our formative evaluation we made some changes in an attempt to improve our materials, and second, we introduced new measures in order to make more specific evaluations. Much more research needs to be carried out in order to disentangle these two hypotheses.

In addition, we should highlight our results regarding health (health-related problems). This variable referred to whether participants reported health-related problems (e.g., back problems). Our prediction was that health would not change in the post-test, with changes only reported in the follow-up. As predicted, in the first and second studies there were no differences between pre-test and post-test in this variable, but in the third study, both the experimental and control groups reported fewer health-related problems in the post-test. These results cannot easily be understood. However, bearing in mind that the Live and Multimedia programs had a duration of 3 months and did not include specific medical care, changes in health could not be expected, since they would only occur as a result of changes in lifestyles: on the other hand, as expected, positive changes in were indeed observed in our first study in the follow-up at 6 months. 

It should also be stressed that the *Vital Aging* programs had only minor impact upon the hypothesized variables related to control (self-efficacy for aging) and social relationships (quality and satisfaction). New analyses have been carried out in order to learn more about these results. Since our participants improved their self-images of aging in both versions—Life and Multimedia (both in the community and in residences)— in our third study we added a new measure of self-efficacy for aging. However, in this study no changes at all were found after the program, though this measure showed a high level of reliability and construct validity [27].

Finally, we must add that we expect to have the summative results from the Vital Ageing e-Learning course available soon, since post-tests have already been administered in the four countries (Spain, Cuba, Mexico, and Chile) involved. From our preliminary results from the subsample in Spain it can be concluded that they are consistent with the other *Vital Aging* versions. 

All of the studies reported here have some important limitations. First of all, our samples are small, and not representative. Our results can be generalized only to those older adults who are *willing* to age well and register in a program for promoting aging well. Second, changes produced refers mainly to immediate changes in behavioral life styles and not long term outcomes such as disability or survival and we carried out only one follow-up study, and the extent of the follow-up was quite limited. We are aware, this Program requires longer follow-up in order to test whether those changes in behaviors could produces effects on long term hard variables such as disability and healthy survival. In the near future, we are planning to follow up all our participants, since 1996, on the *Vital Aging* programs. Third, research on active aging is growing rapidly, so that active aging promotion programs cannot be “closed” in a particular set of units (or contents), since empirical evidence is increasing year on year, and new elements are continually being discovered, supported by empirical or experimental evidence, that can influence positive aging, so that they must be introduced in a flexible way. It is on the basis of this aspect that we have launched an Internet Site which can be updated for providing material to both users and professionals (http://www.vivirconvitalidad.com/). Fourth, as remarked by Fernandez-Ballesteros in a follow-up study on aging stereotypes, the media not only generate negative stereotypes in relation to the aging phenomenon, but can also produce positive changes in the mentality of new generations, embedding positive images about aging in line with the idea that individuals can be agents in their own aging process [64]. Therefore, we are aware that in the future it will be necessary to adjust the content and methodology of *Vital Aging* in accordance with a rapidly changing society—adapting them to generations of older adults who are increasingly demanding, better prepared, and better educated, so that we may need to introduce different levels of difficulty into our program. Finally, on the basis of our first study applied in Residences we have ceased the administration of *Vital Aging* in institutions, but we do believe that much more effort should be made to design a new version that could be implemented in institutions and in other settings.

Aging is an international phenomenon; it is an expression of the human being's capacity for adaptation, or plasticity—at both individual and population levels*—*and also a product of the level of development of our society and of its success. However, aging can also be considered a threat, as it is associated with illness and disability. National, regional and international institutions are calling for the implementation of initiatives, policies, and programs for extending health and well-being across the lifespan and into very old age, converting active aging into a kind of mantra. But active aging (or successful, optimal, productive, and *vital aging*) are also scientific concepts about which a substantial body of knowledge can be disseminated and applied at both the population and individual levels. It should not be overlooked that individuals themselves are the agents of their own development and aging, and the most important resource for change. *Vital aging* represents only a modest step forward in this direction, and this paper is no more than a way station on the long, but fascinating, path in pursuit of better aging, as we try to convince people that, as well as adding years to life, they can always add life to years.

## Figures and Tables

**Table 1:** Summary of *Vital Aging* versions: procedures for implementation and evaluation.

	*Vital Ageing* L	*Vital Ageing* M	*Vital Ageing* e-Learning
Date of implementation	1996–2003	2002–2012	2012

Duration each edition	3 months, 70 hours (3 hours/session; 2 sessions per week)	3 months, 48 hours (2–4 hours per session, 2 sessions per week)	3 months: 1 unit per 2 week

Trainers	Experts	Experts from Germany, Italy, and Spain Organized by a Tutor	Organized by a Virtual Tutor and an On-site tutor

Materials	Basic Text: Fernández-Ballesteros [11] Standard classes	Multimedia learning technology Video Lessons	Basic texts: http://www.vivirconvitalidad.com/ Learning management System, LMS- Moodle Platform

Financed	Institute of Older Adults and Social Services (IMSERSO)	European Commission (Vitalgell-C Project, 2002)	UAM-Santander Inter-University Cooperation Program for Latin America (PUM-e, 2010–2012)

Session procedure	(1) Introduction; (2) Pre-test; (3) Practice and exercises; (4) Post-test; (5) Conclusions and remarks	(1) Introduction; (2) Pre-test; (3) Practice and exercises; (4) Post-test; (5) Conclusions and remarks	(1) Introduction; (2) Pre-test; (3) Readings; (4) Practice and exercise; (5) Forums; (6) Tutorial; (7) Post-test in each unit

Recruitment	Announcements in newspapers, on radio and in UAM promotion systems	Announcements at selected Senior Citizens' Clubs and at UAM, to Students from University Programs for Older Adults	Students from University Programs for Older Adults at the four universities

Participants	240 volunteers attended the program (approximately 35 per course; Mean age = 72.3, range = 57–83, SD = 6.7; 70% women)	155 volunteers (around 10–22 per course; Mean age = 69.9, range = 60–94, SD = 6; 76% women)	88 volunteers: UAM (*N* = 26), La Habana University (*N* = 20), National University Autonomous of Mexico (*N* = 23) and Catholic University of Chile (*N* = 19). (Mean age = 64.2; SD: 7.57, range = 49–84; 84% women)

**Table 2:** Domains, units, contents, and assessment and practice of *Vital aging* versions.

Domains	*Vital aging* L and *Vital aging* M			*Vital aging* e-Learning		
	Units	Contents	Assessment and practice	Units	Contents	Assessment and practice
	Aging well	(i) General introduction to the Course (ii) Human development is lifelong (iii) Use it or lose it! (iv) What vital, successful, active, and productive ageing means: the four domains (v) Mechanisms for aging well: the SOC model (vi) Stereotypes and self-steretoypes of aging	(a) Your images about aging? (b) Which are aging well mechanism? (c) Level of your physical activity? (d) Your social relations? (e) Avoid state “I already cannot”	Aging well	(i) Active aging. Aging well (ii) Stereotypes and self-stereotypes of aging (Myths and realities about aging) (iii) Why “I cannot.”	(a) What is aging well? (b) Active aging versus aging well task (c) How to identify my stereotypes and combat them? (d) How I am getting old? (e) Forum: Blessed versus damn old age
	Enjoy the Control of your life	(i) Importance of healthy lifestyles (ii) The concept of health (iii) How to learn new healthy habits (iv) Misconceptions about health (v) Health Crisis along life course (vi) Risk factors: how to control them (vii) Protective health factors (viii) How to improve self-esteem	(a) Assess what is going well/what can be improved (b) Target behaviours selection for change: (1) Short term (2) Long term	Take care of your body	(i) Physical activity: exercise and sport, its importance, and changes across life (ii) Good nutrition characteristics (iii) Take care of your teeth and your feet	(a) Identifying aging signs (b) How is your fitness? (c) How is your diet? (d) In what extent do you take care of your teeth and feet? (e) Plan your exercise (f) Plan your nutrition changes, and diet (g) Plan how your body caring (h) Forum: You will is you can
Behavioral health and independence	Health and Nutrition: Good food, good life	(i) Nutrition as one of the important aspects for health and aging (ii) Food as energy. Food guide Pyramid (iii) Nutrition fact (iv) How to build a healthy body (v) Changes in diet are required across lifespan (vi) How to cook healthy recipes	(a) Assess your food information (b) How calculate BMI (c) Assess your nutrition (d) Planning food for the next week/month			
	Taking care of your body: Self-responsibility and self-management	(i) Body changes across the lifespan: a trip through the body across time (ii) Your 5 senses: where they are placed (iii) The importance of your teeth and your feet (iv) Self-responsibility to be independent in performing activities of daily living, choosing social contact, and personally meaningful interests (v) Older adults as social capital	(a) Take your mobility test (b) Take your balance test (c) Plan how to improve your body care (d) How to walk without risks (e) How to promote and maintain ADL			
	Regular exercise: the best formula for aging well	(i) Regular physical exercise and activity as one of the best means for ageing well physically and mentally (ii) Benefit of physical exercise at physiological, cognitive, emotional, and social levels (iii) Aerobic, strength, flexibility, and balance	(a) In what extent you exercise? (b) What do you do, what do you need? (c) Selecting physical activity and exercise to incorporate into your daily life (d) Make your plan (e) Assess you base line and following-up			

	Train your mind: how to prevent brain ageing	(i) Change and Stability of cognitive functioning across life span (ii) Solving familiar and unfamiliar problems (iii) Managing everyday tasks (iv) Remember intended activities (v) Effects of cognitive training physical activities and psychological variables in brain functioning	(a) Test your cognitive functioning (b) Proposals of exercises and cognitive activities to train mental abilities and prevent brain ageing (c) Plan your brain training (d) Assess your base-line a continue following-up	Take care of your mind	(i) Cognitive functioning (ii) Change and Stability of cognitive functioning across life span (iii) Selection, optimization and compensation as mechanisms of adaptation to changes	(a) How do you take care of your mind? (b) Select your favourite cognitive activities (c) Self-observation of your mental decline and stability (d) Check what you have learned (e) Plan your cognitive activity (f) Forum: Cognitive functioning among the very old
Cognitive functioning	Improve your memory	(i) Misconceptions about memory and ageing. (ii) What is memory, how is it organized, and how does it work? (iii) Aging effects on memory. Memory problems (iv) How to improve memory through mnemonic	(a) Test your memory (b) Daily self-register of cognitive activity (c) Mnemonic skills training (d) Memory training			
	Wisdom: the expression of lifelong learning	(i) Wisdom: Lay (implicit), explicit, and expert theories (ii) Wisdom development across time (iii) Wisdom: in between intelligence and personality	(a) How you define “wisdom”? (b) Test your wisdom (c) How to train wisdom			
	The creative age	(i) What is creativity? (ii) Old people creativity (iii) Stereotypes about creative behaviour and ageing (iv) How to be creative	(a) Test your creativity (b) Choose preferred activities for expressing creativity			

Affect, control and coping styles	Self-efficacy Perception	(i) Primary and secondary control (ii) Self-efficacy as expression of control (iii) The belief of ageing successfully as predictor of aging well (iv) Self perception of aging	(a) You as a model of aging well (b) Others as modeling (c) Imageries of success (d) Solving life events across lifespan and solving life events in old age	Feel happy	(i) Emotion: pleasant activities and well-being (ii) Control and self-efficacy (iii) Coping with stress	(a) How do you feel? (b) Pleasant activities questionnaire (c) Weekly self-registration activities (d) Self-efficacy scale (e) How do you cope with stress? (f) Plan how to cope with stress
	Positive thinking	(i) We are what we think (ii) Attitudes and thought (iii) Thinking errors (iv) Positive thinking	(a) Test your positive thinking (b) Identify thinking errors (c) Turn negative experiences into positive ones			
	Coping with stress	(i) What is stress and anxiety? (ii) Coping with stress (iii) Active and passive coping (iv) Coping skills across life span	(a) Learn self-instructions, cognitive, emotional, and physiological coping strategies (b) How to apply them			
	Death is also part of life	(i) Life and Death (ii) Bereavement (iii) Spiritual approach (iv) Transcendence (v) Meaning in life	(a) Test your fear to death (b) Think about you death (c) I think, I feel, I do			
	Pleasant activities and well-being	(i) Activity as a source of life (ii) Feeling of depression (iii) Pleasant activities and well-being (iv) Use it or lose it	(a) Test your base line of activity (b) Plan pleasant activities: analysing resources and limitations (c) Plan and Self-monitoring your activity and well-being			

	How to improve relationships with family and friends	(i) Human relationships needs (ii) Family, friends and others: their benefits (iii) Social relationships and independence (iv) Give and received (v) Social skills (vi) Emotional intelligence	(a) Test your social networks (b) How to improve social skills (c) Training empathy, assertiveness, say “no” say “yes” (d) Interpersonal conflict management	Get involved with others	(i) Family (ii) Friends (iii) The others: Social participation	(a) My relationships (b) Assess your social life (c) Friends' network (d) Forum: Spanish grandparents, looking after grandchildren
Social participation and engagement	The others need me too	(i) Importance of pro-social behaviour (ii) Stereotypes of personality changes (iii) Pro-social behaviours and well-being (iv) How to improve care relationships (v) Care and caring (vi) Volunteering	(a) Test your pro-social behaviour (b) Plan pro-social behaviours in common life (c) Train emotional self-control			
	Sexuality: beyond genitality	(i) What is sexuality? (ii) Stereotypes and social pressure in old people sexuality (iii) Sexuality beyond genitals: diverse modalities (iv) Aging and sex: physiological changes	(a) Sensitivity and sexuality (b) Train what you do not see (c) Pelvic floor muscles exercises			
	A new system of communication: Internet	(i) Healthy behaviours in computer used (ii) Stereotypes of old people using computers (iii) Computers for hobbies, communication, navigation, and so forth	(a) How to use computers, Internet, and its different applications (b) All practice			

Fernández-Ballesteros, 2002 [12] (5 Volumes); http://www.vivirconvitalidad.com/.

**Table 3:** Sample Characteristic of the four studies curried out.

Studies	Participants	*N *	Mean age
(1) *Vital Aging* M	Community	44	69.9
	Residential	13	79.3
	Control	31	74.2

(2) *Vital aging* M versus *Vital aging* L	*Vital Aging* M	25	69.5
	*Vital aging* L	28	67.84
	Control	37	65.6

(3) *Vital Aging* M	*Vital Aging* M	73	62.56
	Control	42	62.29

(4) *Vital Aging* e-Learning	*Vital Aging* e-Learning	88	64.2
	Control	42	62.29

## References

[1] Roth M (1960). Problems of an ageing population. *British Medical Journal*.

[2] Fries JF, Crapo LM (1981). *Vitality and Aging*.

[3] Fries JF (1980). Aging, natural death, and the comprenssion of morbidity. *The New England Journal of Medicine*.

[4] Fries JF (1989). *Aging Well*.

[5] Christensen K, Doblhammer G, Rau R, Vaupel JW (2009). Ageing populations: the challenges a head. *The Lancet*.

[6] Vaupel JW (2010). Biodemography of human ageing. *Nature*.

[7] Baltes PB, Baltes MM, Baltes PB, Baltes MM (1990). Psychological perspectives on successful aging: the model of selective optimization with compensation. *Successful Aging: Perspectives from the Behavioural Sciences*.

[8] Schaie KW (2005). What can we learn from longitudinal studies of adult development?. *Research on Human Development*.

[9] WHO (2002). *Active Ageing*.

[10] Fernández-Ballesteros R (2008). *Active Aging. The Contribution of Psychology*.

[11] Fernández-Ballesteros R, Carretero M (1986). Hacia una vejez competente: Un reto para la ciencia y la sociedad. (Toward a competent aging. A challenge for science and society). *Psicología Evolutiva*.

[12] Fernández-Ballesteros R (2002). *Vivir con Vitalidad (Vital Aging) *.

[13] Fernández-Ballesteros R (2005). Evaluation of “Vital Aging-M”: a psychosocial program for promoting optimal aging. *European Psychologist*.

[14] Caprara MG (2005). *Envejecimiento con éxito: valoración de un programa [Successfulaging: evaluation of a program] [Ph.D. thesis]*.

[15] Fernández-Ballesteros R, Caprara MG, García LF (2004). Vivir con Vitalidad-M: un Programa Europeo Multimedia. *Intervención Social*.

[16] Fernández-Ballesteros R, Caprara MG, Iñiguez J, García LL (2005). Vivir con Vitalidad-M. A European multimedia programme. *Psychology in Spain*.

[17] Capara MG, Fernández-Ballesteros R (2012). *Promoting Active Aging: New Effects of Vital Aging Program*.

[18] Rowe JW, Kahn RL (1987). Human aging: usual and successful. *Science*.

[19] Bandura A (1997). *Self-Efficacy: The Exercise of Control*.

[20] Fernández-Ballesteros R, Botella J, Zamarrón MD (2012). Cognitive plasticity in normal and pathological aging. *Clinical Intervention on Aging*.

[21] Pascual-Leone A, Freitas C, Oberman L (2011). Characterizing brain cortical plasticity and network dynamics across the age-span in health and disease with TMS-EEG and TMS-fMRI. *Brain Topography*.

[22] Fernández-Ballesteros R, Lage M (2002). Envejecimiento satisfactorio, (Satisfactory aging). *Corazón Y Cerebro, Ecuación Crucial De Envejecimiento (Heart and Brain and Equation of Aging)*.

[23] Fernández-Ballesteros R, Schettini R, Molina MA, Santacreu M (2012). *Testing a Four Domain Model of Aging Well*.

[24] Fernández-Ballesteros R, García LF, Abarca D (2008). Lay concept of aging well: cross-cultural comparisons. *Journal of the American Geriatrics Society*.

[25] Fernández-Ballesteros R, García LF, Abarca D (2010). The concept of “ageing well” in ten Latin American and European countries. *Ageing & Society*.

[26] Fernández-Ballesteros R, Molina MA, Schettini R, Santacreu M, Robine JM, Jagger C, Crimmins EM (2013). The semantic network of aging well. *Healthy Longevity*.

[27] Fernández-Ballesteros R, Zamarrón MD, Diez-Nicolás J (2010). Envejecer con éxito. Criterios y predictores. *Psicothema*.

[28] Bogers RP, Tijuis MR, Van Gelder BM, Kromhout D (2006). *Final Report of the HALE (Healthy Aging: A Longitudinal Study in Europe Project)*.

[29] Haveman-Nies A, de Groot LCPGM, van Staveren WA (2003). Dietary quality, lifestyle factors and healthy ageing in Europe: the SENECA study. *Age and Ageing*.

[30] Fries JF (2002). Reducing disability in older age. *Journal of the American Medical Association*.

[31] Netz Y, Wu MJ, Becker BJ, Tenenbaum G (2005). Physical activity and psychological well-being in advanced age: a meta-analysis of intervention studies. *Psychology and Aging*.

[32] Sofi F, Cesari F, Abbate R, Gensini GF, Casini A (2008). Adherence to Mediterranean diet and health status: meta-analysis. *British Medical Journal*.

[33] Trichopoulou A, Orfanos P, Norat T, Bueno-de-Mesquita B (2005). Modified Mediterranean diet and survival: EPIC-elderly prospective cohort study. *British Medical Journal*.

[34] Trichopoulou A, Vasilopoulou E (2000). Mediterranean diet and longevity. *British Journal of Nutrition*.

[35] Knoops KT, de Groot LC, Kromhout D, Horng MS (2004). Healthy lifestyle and mediterranean diet decreases mortality in the elderly. *Journal of Clinical Outcomes Management*.

[36] Bernis C (2007). Gender, reproductive ageing, adiposity, fat distribution and cardiovascular risk factors in spanish women aged 45–65. *Anthropologist Special*.

[37] Wilson RS, Barnes LL, Bennett DA (2003). Assessment of lifetime participation in cognitively stimulating activities. *Journal of Clinical and Experimental Neuropsychology*.

[38] Verghese J, Lipton RB, Katz MJ (2003). Leisure activities and the risk of dementia in the elderly. *New England Journal of Medicine*.

[39] Ball K, Berch DB, Helmers KF (2002). Effects of cognitive training interventions with older adults: a randomized controlled trial. *Journal of the American Medical Association*.

[40] Verhaeghen P (2000). *The Interplay of Growth and Decline: Theoretical and Empirical Aspects of Plasticity of Intellectual and Memory Performance in Normal Old Age*.

[41] Hertzog C, Kramer AF, Wilson RS, Lindenberger U (2009). Fit Body, Fit Mind?. *Scientific American Mind*.

[42] Colcombe S, Kramer AF (2003). Fitness effects on the cognitive function of older adults: a meta-analytic study. *Psychological Science*.

[43] Pressman SD, Cohen S (2005). Does positive affect influence health?. *Psychological Bulletin*.

[44] Ostir GV, Ottenbacher KJ, Markides KS (2004). Onset of frailty in older adults and the protective role of positive affect. *Psychology and Aging*.

[45] Staudinger UM, Freund AM, Linden M, Maas I, Baltes PB, Mayer KU (1999). Self, personality, and life regulation: facets of psychological resilience in old age. *The Berlin Aging Study: Aging from 70 to 100*.

[46] Labouvie-Vief G, Johnson ML (2005). The psychology of emotions and ageing. *The Cambridge Handbook of Age and Ageing*.

[47] Lachman ME (2006). Perceived control over aging-related declines: adaptive beliefs and behaviors. *Current Directions in Psychological Science*.

[48] Lachman ME, Andreoletti C (2006). Strategy use mediates the relationship between control beliefs and memory performance for middle-aged and older adults. *Journals of Gerontology B*.

[49] Lachman ME, Firth KM, Brim OG, Ryff CD, Kessler R (2004). The adaptive value of feeling in control during midlife. *How Healthy Are We? A National Study of Well-Being at Midlife*.

[50] Levy BR, Slade MD, Kunkel SR, Kasl SV (2002). Longevity increased by positive self-perceptions of aging. *Journal of Personality and Social Psychology*.

[51] Wurm S, Tesch-Römer C, Tomasik MJ (2007). Longitudinal findings on aging-related cognitions, control beliefs, and health in later life. *Journals of Gerontology B*.

[52] Morrow-Howell N, Hinterlong J, Rozario PA, Tang F (2003). Effects of volunteering on the well-being of older adults. *Journals of Gerontology B*.

[53] Mendes de Leon CF, Glass TA, Berkman LF (2003). Social engagement and disability in a community population of older adults: the New Haven EPESE. *American Journal of Epidemiology*.

[54] Park DC, Gutchess AH, Meade ML, Stine-Morrow EA (2007). Improving cognitive function in older adults: nontraditional approaches. *The Journals of Gerontology B*.

[55] Zunzunegui MV, Rodriguez-Laso A, Otero A (2005). Disability and social ties: comparative findings of the CLESA study. *European Journal of Ageing*.

[56] Scriven M (1991). *Evaluation Thesaurus Edition*.

[57] Millsap RE, Maydeu-Olivares A (2009). *The Sage Handbook of Quantitative Methods in Psychology*.

[58] ADA (2005). Position paper of the American Dietetic Association: nutrition across the spectrum of aging. *Journal of the American Dietetic Association*.

[59] LIFE Study Investigators (2006). Effects of physical activity intervention on measures of Physical Performance: results of Life styles Interventions and Independence for Elders Pilot (LIFE-P) Study. *Journal of Gerontology*.

[60] Dwyer JT, Coleman KA, Krall E (1987). Changes in relative weight among institutionalized elderly adults. *Journals of Gerontology*.

[61] Fernández-Ballesteros R, Caprara G, Schettini R Effects of University Programs for Older Adults. Changes in cultural and group stereotypes, self-perception of aging, and emotional balance.

[62] Fernández-Ballesteros R, Molina MA, Scgettini R, del Rey AL (2012). Promoting active aging through University Programs for Older Adults: an evaluation study. *Geropsychology*.

[63] Diener E (1999). Introduction to the special section on the structure of emotion. *Journal of Personality and Social Psychology*.

[64] Fernández-Ballesteros R (2006). GeroPsychology an applied field for the 21st century. *European Psychologist*.

